# Applying the Surge Capacity Components for Capacity-Building Purposes in the Context of the EMT Initiative

**DOI:** 10.3390/ijerph21121712

**Published:** 2024-12-23

**Authors:** Lina Echeverri, Flavio Salio, Richard Parker, Pryanka Relan, Oleg Storozhenko, Ives Hubloue, Luca Ragazzoni

**Affiliations:** 1 CRIMEDIM (Center for Research and Training in Disaster Medicine, Humanitarian Aid and Global Health), Università del Piemonte Orientale, 13100 Vercelli, Italy; luca.ragazzoni@uniupo.it; 2 World Health Organization, 1211 Geneva, Switzerland; saliof@who.int (F.S.); relanp@who.int (P.R.); 3 Training in Aid, QLA 2130 Qala, Malta; rich@traininginaid.com; 4 World Health Organization, EURO Regional Office, DK-2100 Copenhagen, Denmark; storozhenkoo@who.int; 5 REGEDIM (Research Group on Emergency and Disaster Medicine), Vrije Universiteit, 101 Jette Brussel, Belgium; ives.hubloue@uzbrussel.be

**Keywords:** capability building, capacity building, disaster preparedness, emergency medical teams, health systems resilience, surge capability, surge capacity, readiness and response

## Abstract

Background: On 16 January 2021 (EB148/18 Session), the World Health Organization (WHO) and Member States emphasized the importance of expanding the WHO Emergency Medical Teams (EMT) Initiative, investing in a global health workforce and multidisciplinary teams capable of being rapidly deployed, equipped, and fully trained to respond to all-hazard emergencies effectively. This resulted in the need to define a comprehensive framework. To achieve this, the EMT Initiative proposes the application of the four components of Surge Capacity, known as the 4“S” (Staff, Systems, Supplies, and Structure/Space), to build global capacities and capabilities, ensuring rapid mobilization and efficient coordination of national and international medical teams for readiness and response, complying with crisis standards of care defined in an ethical and evidence-based manner. Methods*:* A mixed-qualitative research approach was used, incorporating expert consensus through focus group discussions (FGDs), between 2021 and July 2022. This facilitated a detailed process analysis for the application of the surge capacity components to build global capacities and capabilities. This research highlighted the similarities between surge capacity and capacity building from an initial desk review and unified these concepts within the EMT Initiative. A standardized formal pathway was developed to enhance local, regional, and global capacities for emergency readiness and response. Results*:* The results showed that the framework successfully integrated the essential components of surge capacity and capacity building, making it adaptable to various settings. Conclusions: This framework provides a unified and replicable approach for readiness and response for all-hazards emergencies.

## 1. Introduction

Disasters are significant disruptions to a community caused by hazardous events and their interaction with vulnerabilities and exposure. These disruptions exceed the capacity of the affected society or community to manage the event using its own resources (UNDRR, IFRC) [1,2]. Disasters and health-related emergencies are occurring with increasing frequency, severity, and complexity. During the last 50 years, the number of disasters has increased fivefold, largely driven by climate change (UNFCCC) [3]. In 2022 alone, there was a rapid emergence and re-emergence of epidemic-prone diseases, worsening ecological degradation, escalating geopolitical conflicts, and the weakening of health systems (WHO) [4]. As of mid-2024, 165,273 political-violence events have been recorded, reflecting a 64% surge in conflicts between 2020 and 2024. These include ongoing conflicts in Ukraine, Palestine, Myanmar, and Sudan, as well as numerous violent events in other countries. Additionally, there has been a significant rise in the involvement of armed groups in these events (ACLED) [5]. All of these, combined with slow progress in health and social indicators in low-and-middle-income countries (LMIC), along with global economic and financial imbalances, are exacerbating health disparities and straining health systems [6]. The convergence of these factors, along with the COVID-19 pandemic, has demonstrated that the world remains underprepared to predict, prevent, respond to, and recover from multi-country outbreaks and other disasters [7]. This situation underscores the urgent need for more flexible and multidisciplinary teams capable of rapidly adapting to any context and repurposing as necessary. It also emphasizes the importance of investing and strengthening local capacities.

The World Health Organization (WHO) Emergency Medical Teams (EMTs) Initiative aligns with the International Health Regulations (IHR 2005), which call on Member States to develop public health capacities to “respond promptly and effectively to public health risks and public health emergencies of international concern” [8]. In 2020, the WHO Executive Board Resolution (EB 146.R10) [9] called upon Member States, international, regional, and national partners and donors to strengthen the role of the local health and allied workforce and to develop an effective and high-performing, national, subnational and regional Emergency Medical Teams, in line with the WHO classification and minimum standards (EMT Initiative-Blue Book 2nd Edition) [10]. This was reiterated in 2021, during the 148th Executive Board Session (EB148/18, 16 January 2021), where commitments to invest in a global health workforce were made, building rapidly deployable multidisciplinary teams fully trained and equipped to respond to all-hazard emergencies [10].

Notwithstanding the many commitments, it is still widely acknowledged the slow progression in building local capacities. Essential components such as strengthening partnerships and networks, ensuring knowledge sharing and skills transfer, long-term funding, and bridging the gap between humanitarian and development activities are much needed if local institutions are expected to take the lead and be better prepared to respond to any health emergencies [11].

### The Terms Capacity Building and Surge Capacity and Their Relevance to Health Emergency Readiness and Response

The term capacity building encompasses various definitions. It involves the process of developing and strengthening the skills, instincts, and abilities of communities to adapt and thrive [12] and the enhancement of knowledge, management skills, and other capabilities through technology and training [13]. However, capacity building is often misinterpreted and mistakenly equated to training. Capacity building is a broader, abstract, and multidimensional concept [14] that refers to a wide range of activities designed to improve or enhance an organization’s or community’s ability to absorb change effectively and sustain itself efficiently over time [15].

Capacity building has a place in the disaster-management cycle, particularly in determining the creation of specific capabilities and supporting the decision-making process regarding their use and localization. Normally, in disaster management, building local capacity is seen to be a pre-event added value, with activities targeting mitigation, preparedness, early warning systems, and strengthening of relief workers’ skills [11].

Conversely, the umbrella term “surge capacity” shows substantial variation in concepts, definitions, and applications [16]. In disaster medicine, surge capacity has been linked to the ability to cope with a sudden and unexpected increase in the number of patients that exceeds the normal operational capacity of the affected facility or health service/system. In humanitarian contexts, the term is often related to the ability of an agency to scale up rapidly and effectively to meet the increase in demands of an affected population, indicating the importance of developing scalable components and the need for continuous investment if agencies are to respond rapidly and effectively when needed [17].

Considering the above-mentioned concepts and information, the aim of this study is to apply the surge capacity components commonly referred to as the “4’S” (Staff, Structure/Space, Systems, and Supplies) in the context of the Emergency Medical Teams Initiative. It is focused on the following two specific objectives:

1- Integrate the four-tier hierarchy capacity-building pyramid with the 4“S” components of the surge capacity.
2- Develop the capacity-building framework for the Emergency Medical Teams Initiative using the surge capacity components.

## 2. Materials and Methods

A mix of qualitative research methods and data collection techniques in three phases, from June 2021 to July 2022, integrating an expert-consensus methodology in the form of focus group discussions (FGDs), were used to support the application of the surge capacity components known as the 4“S” to build the framework for capacity-building purposes within the context of the EMT Initiative.

An initial desk review (first phase) supported the process. The purpose of the review was to obtain a thorough understanding of the use of the surge capacity components for capacity-building efforts in the healthcare domain, including preparedness, readiness, and response to health emergencies.

The review search strategy was guided by the hypothesis that enhancing surge capacity supports readiness and rapid deployment of EMTs (Emergency Medical Teams), enabling Member States and organizations to mitigate the impact of health emergencies.

The desk review used both peer-reviewed literature and grey literature. The review was conducted from June 2021 to July 2021. The review of the literature went as far as necessary to find literature directly relevant to the research aim and objectives.

Peer-reviewed articles were extracted and collected from different databases (ELSEVIER, PLOS, PubMed, The Milbank Quarterly, Google Scholar, and ResearchGate), and a grey literature (such as policy documents, reports, and guidelines) targeted search on organizations websites (WHO, ALNAP, ACLED, UN, IFRC) complemented the review. Keywords included the following: surge capacity, capacity building in healthcare, disaster preparedness, readiness and response, health emergencies, emergency medical teams, and health system resilience. The rationale for using the surge capacity components lies in two facts: first, the surge capacity has been seen by the EMT Initiative as the main component of healthcare-system readiness and proposed for capacity strengthening, particularly, but not only, at the national level [8]; and second, surge capacity has been used in disaster and emergency medicine to plan for an increased influx of patients, specialized care requirements and to increase the ability of the healthcare system to cope with changes in demand [18].

A small subject matter-experts group discussion was convened during this first phase. Before the discussion, the participants were acquainted with the aim and objectives of the study. For the purpose of this group discussion, an expert [19] is considered to have a high level of knowledge or skills pertinent to the task; here specifically, the participants of this group were selected based on their competencies and extensive experience in areas comprising disaster medicine and management, design, development, and delivery of capacity building activities, research methods, and data collection and analysis related to experts’ opinions exercises from a variety of health domains.

This group of five experts was tasked to (a) support the revision of the articles gathered from the desk review, (b) perform a thematic analysis to identify patterns within the literature regarding the previous use of the surge capacity components for capacity-building activities, and (c) work on the initial conceptualization (concept paper) of the preliminary framework, which comprised the scope, rationale, objectives, and expected outcomes to be further validated in the second phase. The group followed a structured and iterative approach focusing on specific tasks, as described above. The experts converged on the selection of the articles that fit the purpose of supporting the development of the capacity-building framework for the EMT Initiative using the surge capacity components. Consensus was built through open dialogue iterated on common ideas.

The second phase consisted of three parallel FGDs [20] involving a selected group of experts from the EMT global network. As written above, experts, by definition, were considered to have a high level of knowledge or skills pertinent to the task. For these FGDs, the participants were selected based on their institutional roles, backgrounds and/or expertise in domains such as health policy, strategy, research, training, and extensive experience in field operations (Table A1), and all of them had expertise and extensive knowledge of the EMT Initiative processes, standards of care, and development of teams. The objective of these FGDs was to discuss the result of the thematic analysis of the desk review and seek consensus on the use of the surge capacity components for the conceptualization of the capacity building framework, in addition to gathering relevant information and recommendations to improve the quality, usability, implementation, and validation of the framework.

Participants of these focus group discussions were invited to participate via email and received brief documentation containing a clear explanation of the objective of the study and the preliminary version of the framework, as well as specific instructions for FGD participation. A participatory approach was adopted, and discussions were held in English. At the start of each of the three FGDs, the participants were walked through a PowerPoint presentation where the concepts of capacity building and surge capacity were presented, the results of the desk review were summarized, and the preliminary version of the framework was introduced. The participants were asked to respond to four questions, as shown in Table 1.

The discussions during these focus groups were structured around the thematic analysis from the desk review, thorough open-ended questions, and guided discussions that facilitated in-depth exploration of the topics. The participants provided qualitative feedback on the conceptualization, usability, development, and implementation of the capacity-building framework.

Data from these discussions were systematically recorded using Excel sheets for later analysis. Consensus was reached through open dialogue and deliberation. Final data interpretation and analysis were performed using method triangulation [21] of all combined methods used during the study, the cross-referenced findings from the desk review, the thematic analysis, and the consensus from the FGDs, to ensure convergence, solidification, and validation of the concepts drawn from the first phase to the three group discussions, and finally, the definition of a comprehensive framework with actionable recommendations.

**Figure 1:**
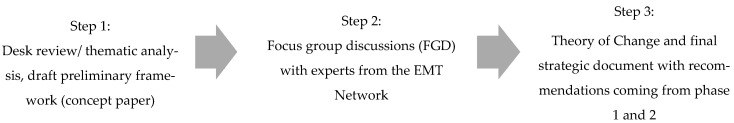
Research steps.

The final product, emerging from all three steps (Figure 1) combined, was a written document presenting the capacity-building framework, including a logical framework to support monitoring, implementation, and evaluation of the activities. The product of these consultations was later presented at the WHO EMT Global Meeting held in Armenia in October 2022 [22].

## 3. Results

### 3.1. Desk Review

The thematic analysis of the desk review showed similarities between the surge capacity and capacity building terms and helped determine if the surge capacity components have been used before to support capacity building activities in the preparedness, readiness, and response to health emergencies.

The literature shows that overall systems for building global-disaster risk management capacity lack a systematic and strategic approach across countries. Past evaluations have shown that agencies are failing to build local capacity and over-rely on international staff to fulfill tasks that could be managed more efficiently by local staff and organizations [11]. Furthermore, capacity building has been a component of organizations’ exit strategies, where a variety of health services and facilities are frequently “handed over” to local authorities without considering their capacity to manage and supply them over time [11].

Potter and Brough (2004) [23] emphasized the importance of systematic capacity building to improve project/program design, execution, and monitoring of programs, ensuring a better use of resources, which translates into developing a sustainable and robust system. For this purpose, they described a pyramid of separate but interdependent levels/components organized in a hierarchical way, showing how the effectiveness of one depends on the effectiveness of others. These components formed what they called a “four-tier hierarchy” of capacity-building needs: (a) structures, systems, and roles; (b) staff and facilities/infrastructure; (c) skills; and (d) tools.

Capacity building has been, therefore, the objective of many development programs [23], but lately and due to the increase of all types of health emergencies around the world, its mention, reference, and use in many disasters and emergency preparedness and response plans by non-governmental organizations (NGOs), government institutions, and UN agencies are observed independent of its definition, understanding, and interpretation. Moreover, confusion in understanding the difference between capacity and capability building has been observed.

Coincidently, the terms “*medical surge*”, “*hospital surge capacity*”, “*healthcare facility-based surge capacity*”, and “*surge capacity*” in general have been known and used in military casualty care and in emergency and disaster medicine. In these medical fields, it has been proposed as a tool and the cornerstone for the planning of activities at pre-hospital and emergency departments [24], particularly in mass casualty situations with high numbers of patients, with the objective to reduce morbidity and mortality while maximizing the use of available resources.

The literature, however, shows the lack of a commonly accepted definition for surge capacity and capacity building (Table A2 and Table A3). Hick et al. (2004) [25] described the areas where surge capacity might be required during disasters (public health, healthcare facilities, and community-based patients) and defined surge capability as the specialized resources needed for an appropriate surge capacity. They emphasized that all those terms should comprise structure, medical and ancillary staff, and supplies, but their planning should be modular. Subsequently, Barbisch and Koening (2006) [26] described the four domains or the multi-component approach of the surge capacity, the four (4“S”), similar to the four-tier hierarchy of Potter and Borough, for a successful preparedness and response to disasters and pandemic-induced surge. The 4“S” are systems (integrated policies and procedures), space (structure/facilities), staff (appropriately trained personnel), and supplies and equipment (Watson, Rudge, and Coker) [16]. These four components (4“S”) or domains of the surge capacity have thus far a general agreement within the disaster and emergency medicine specialties (Lavonne M. Adams 2009) [24].

Despite the agreement on the components of the surge capacity, the lack of an agreed definition prevents conceptual clarity and challenges its study. Particularly, its application and measurement by healthcare providers or deployable medical teams remains a challenge when planning, preparing for, or responding to large-scale or complex events [16,24]. Consequently, it makes it even harder to monitor and evaluate its adequacy and effectiveness.

Many articles have been published in the areas covering medical and hospital surge capacity and capacity-building activities for disaster preparedness and response, although no literature was found that established any single set of definitions as standard, any documented similarity between the terms surge capacity and capacity building, nor any literature that proposes the use of the four components (4“S”) for the development and implementation of capacity-building activities.

We, however, consider five articles (Table 2) to be the pillars for our discussion, for their important contributions in trying to find common ground for surge capacity and capacity building, for acknowledging the difference between capacities and capabilities, and for bringing these concepts to the core of any strategy design, all-hazards preparedness, and response plans, and ultimately, for their contribution in paving the way forward towards this framework.

### 3.2. Small Subject Matter-Expert Group Discussion

Discussions in this group were structured around the thematic analysis of the surge capacity components and their use for capacity-building efforts. The participants provided qualitative feedback on the conceptualization, the similarities of both terms, and the usability of the surge capacity components for the development of the capacity-building framework. The thematic analysis identified recurring terms related to surge capacity and capacity building for healthcare preparedness and response. This analysis highlighted the importance of the following themes in the desk review phase: *surge capacity*, central to healthcare system readiness and disaster response; *capacity building*, for the strengthening of healthcare systems, particularly at the national level; *preparedness*, *readiness*, *and response*, core elements of health emergency preparedness and response; and *health systems resilience*, the adaptability to increased demands during emergencies.

The thematic analysis supported the agreement among participants on the fact that the four-tier hierarchy of the capacity building pyramid and the surge capacity components share common components. Table 3 aligns two frameworks: the capacity building pyramid (four-tier hierarchy) and the surge capacity components. The intersection or the *merge* (marked “X”) indicates where the components from the surge capacity and the capacity building align.

To facilitate the understanding of the similarities shown in Table 3, some observations can be made. For example, *structure and staff*/*infrastructure* reflect the need for organizational frameworks and, at the same time, physical infrastructure; the intersection of *staff and skills* denotes the importance of human resources as well as their competencies and expertise, pivotal for ensuring high-quality healthcare delivery; *systems and systems*/*roles*, suggest a focus on governance, operational systems, standards of care, and the definition of roles to ensure consistent coordination during emergency responses; and finally, *supplies and tools* highlight the need for medical supplies, equipment, and other logistic components, foundational for both frameworks and in any given response.

Attempts to unify concepts regarding surge capacity and capacity building were made (Table 4), considering their applicability to the EMT Initiative in the context of readiness and response, and built upon the information gathered through the desk review. These proposed concepts were posteriorly presented to the three FGDs and in the final document of the capacity-building framework. Furthermore, these suggested concepts have been used and led to the definitions adopted by the EMT 2030 Strategy [27]

**Figure 2:**
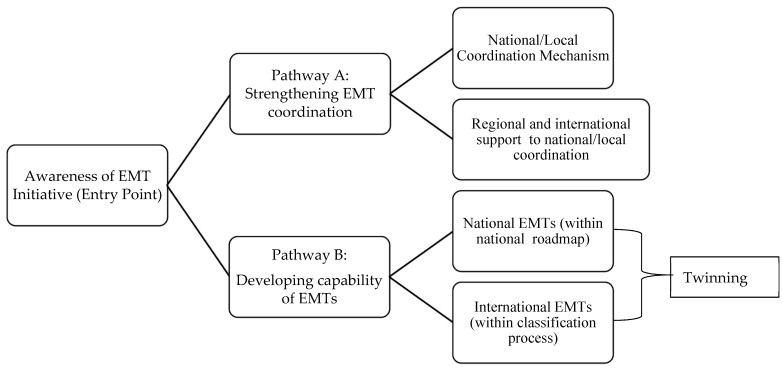
Implementation modalities/pathways.

At the same time, a pathway for the potential implementation of the EMT capacity-building framework was designed and agreed as represented in Figure 2, where:

*Awareness* of the EMT Initiative refers to the “entry point”, which can take a variety of forms. It can be a workshop or a face-to-face introductory event in a way to bring together decision-makers from a country’s health authorities alongside key national or international actors to explore how the EMT Initiative can support the country’s healthcare system or to introduce organizations to the benefits of the EMT Initiative.

*Pathway A* focuses on efforts to enhance the national mechanisms, methodology, and competencies necessary to activate the support of EMTs and to integrate their temporary deployment, from activation to deactivation, within the affected healthcare system. Additionally, pathway A focuses on efforts to strengthen other mechanisms at the regional and/or global level to enable the amplification of requests for assistance of international EMTs. In the broader idea of surge capacity and planning, the development and deployment of EMTs can be seen as additional expertise and resources to bolster a healthcare workforce at different levels of care or to support the network of service providers, including new sites. 

*Pathway B* refers to the collective competencies, expertise, and resources of an EMT to deliver on its deployment mandate according to the specified typology. For national EMTs, a roadmap is envisioned consistent with national norms and accreditation requirements, using as a reference the EMT Classification and Minimum Standards of care [8]. For international EMTs, the standards are mandatory for compliance with each step within the classification process. For this pathway, the twinning project provides a particular modality to ensure mutual exchange of experiences and to reinforce cooperation and support during emergencies.

Finally, building on the similarities of the capacity building and surge capacity components and the pathways for the implementation of the activities, a concept document was written and shared with the participants of the FGDs.

### 3.3. Focus Group Discussions

Before the start of each of the FGDs, participants were acquainted with the components of the capacity building pyramid and the components of the surge capacity. The similarities between these concepts were explained, as well as the potential advantages in using the merge of their components for strategy development, adaptation of policies, and conformation of teams.

An initial assessment showed discrepancies among FGD participants in their understanding of what is and what entails capacity and capability-building activities, and the same regarding the concept (or the multiple concepts) of surge capacity. These discrepancies mirror what we found in the desk review and what other researchers have described thus far.

Notwithstanding the divergence in the understanding of the capacity building and surge capacity concepts, an agreement was reached among participants after the consultations on the use of the four components of the surge capacity for the implementation of the EMT capacity-building framework, and major recommendations emerged from this process, which we summarize in the following points:

(a) Capacity building should be seen as a comprehensive framework that requires a participatory approach for the implementation of activities, particularly peer-to-peer support at the community level.
(b) Constant re-evaluation of activities, anchored on lessons learned from past responses and evidence-based through operational research activities.
(c) Improve coordination and collaboration with other response mechanisms and stakeholders working on disaster resilience, preparedness, and response.
(d) Teams should be agile and flexible enough to repurpose and to adapt to changing needs and environments.
(e) Training pathways should reflect point four and should target peer-to-peer support and on-the-job training and foster knowledge sharing, looking at building long-term sustainable relationships and better defining and improving competencies of local health workforce.
(f) Structures should be modular and flexible to adapt to context needs, enhancing the local resilience of health services.
(g) Ensure funding mechanisms that guarantee the availability and allocation of resources to support the constitution and reinforcement of national capacities and capabilities.

Building on the triangulation method, the process analysis, including the information gathered from the desk review and the consultative process from the FGDs, the following framework (Table 5) has been proposed to strengthen capacity and capability within the EMT network. Table 5 shows the categorization of capacity building levels, national (national EMTs) and international, identifying key activities at each level and transversal to both.

Table 5 was developed as a collaborative framework to integrate the surge capacity components into the capacity-building efforts. For example, the national level focuses on the development of teams based on the adaptation of available standards to context needs with tailored training, whereas international capacities envision compliance with international standards and classification processes. Collaboration amongst these capacities is envisioned through technical training, twinning projects, knowledge-sharing activities, and joint response efforts, to name a few. The transversal activities in the table act as a bridge, providing foundational support for both national and international teams, collaboration, and continuous cooperation. This is expected to improve readiness and response efforts through the utilization of the surge capacity components, ensuring localized and global readiness for health emergencies.

The third phase consisted in the design of a Theory of Change (TOC) a posteriori of the FGDs. This TOC (Table A4) provides a structure to visualize the process and improve planning, monitoring, and evaluation of activities or interventions, which ultimately will support the development and enhancement of global capacity and capability for readiness and response to health emergencies.

## 4. Discussion and Recommendations

We are witnessing how, in the immediate aftermath of a disaster, the demand for local and institutional capacities increases substantially. However, there is little formal empirical research addressing capacity-building interventions for disaster risk management and even poorer research analyzing capacity building during response, resulting in limited evidence to guide strategy for international and local actors and institutions [28].

This research proposes an innovative approach by applying the surge capacity components to build capacity within the context of the EMT Initiative through a consensus-based approach. This is expected to enhance readiness and response capacities while facilitating meaningful dialogue and interaction between the different stakeholders and medical teams within the EMT network, the communities they serve, and ultimately, to influence policies across different areas of work.

The triangulation method used in this study, by integrating data from diverse sources, employing multiple methods, and incorporating various theoretical perspectives, ensures that the capacity-building framework is robust, validated, and applicable across different contexts.

The process of establishing the consensus on the use of the surge capacity components highlighted the willingness and the commitment of the EMT Initiative to self-assess its relevance and how it fits the purpose of leading the improvement of global emergency preparedness, readiness, and response capacities through an evidence-based approach. Furthermore, as highlighted in the newly launched EMT 2030 Strategy [27], it reiterates the need to expand standardization and quality-assurance processes to current thematic areas, emphasizing the potential to adapt the EMT methodology to other response capacities, initiatives, or global programs.

Important recommendations came from this research, such as the modalities to be used by governments and organizations to improve the planning and allocation of resources based on context needs, sustain expansion, and fundamentally, achieve better collective outcomes. These foreseen outcomes are (a) the strengthening and improvement of emergency preparedness, readiness, and response at the local, national, and international level, (b) the fostering of global capacity and capability ensuring compliance with EMTs standards and the implementation of the surge capacity components, (c) the efficient coordination and rapid mobilization, and, finally, (d) the definition and enhancement of skills and competencies of the global health workforce.

In addition to what has been discussed above, important findings can be summarized as follows:

*First*, an agreed definition of surge capacity is missing in the disaster medicine literature and is urgently needed. The lack of a common definition not only interferes with the general understanding of the term surge capacity or prevents its measurement and evaluation (Watson, Rudge, and Cork) [16], (Lavonne M. Adams 2009) [24], but it interferes with the extrapolation and use of its components for disaster readiness and response plans outside of the emergency and pre-hospital settings where the term originated. Nevertheless, the consensus approach used in this study allowed for a unified definition of surge capacity as described in the EMT 2030 Strategy. We believe the reach and impact of the use of the surge capacity components might potentially be larger, particularly for the development of national and regional rapid-response capacities.

*Second*, major similarities exist between the components of the surge capacity and the capacity building four-tier hierarchy of needs proposed by Potter and Brough in 2004 [23]. In this regard, it is the first time that an attempt is made to use the surge capacity components as pillars to define and implement a capacity building framework in relation to disaster readiness and response. We believe this research provides an important innovative contribution to the resilience and adaptation of health systems and to the disaster medicine field.

We argue that the merge of the components presented in this paper will facilitate the understanding, design, and implementation of any response plan for all-hazard events. The surge capacity domains/components must align with a strategic vision built on risk and context analysis and with a structured system of policies and procedures that facilitate the execution of activities.

*Third*, health systems resilience encompasses the strengthening of medical team capacities and capabilities in a way for them to be ready to cope and respond to disasters and other types of health emergencies. Surge capacity, in the terms defined in this paper, plays a pivotal role [24] in facilitating this course of action. The planning for this change and adaptation of health systems requires, however, an iterative process that continues over time [23].

*Fourth*, additional efforts should be made to advance the evaluation of capacity and capability-building activities to inform evidence-based strategies and encourage research from low- and middle-income countries and fragile and conflict-affected settings. Reassess learning objectives and best practices of available training in a way that fits the purpose of EMT members and the overall emergency healthcare workforce, ensuring their adaptation to rapidly changing environments.

Finally, through the adoption of this framework, it is expected that the EMT Initiative will boost the required political will and further harmonize activities across countries and the EMT network. It recommends activities that are framed to enhance health emergency preparedness and readiness and improve the management and development of medical teams. Furthermore, it contributes to the context-based adaptation of services and the efficient coordination of both national and international medical teams while ensuring compliance with the standards of care defined by the EMT Initiative and the enhancement of skills and competencies of the global health workforce.

## 5. Limitations

This study has some limitations. First, the focus group discussions were conducted online, which might hamper interaction. Second, discussions were held in English, and although all participants spoke the language, not all of them had it as their mother tongue, which might have compromised their ability to express ideas, interact better, and share their knowledge. Third, due to time constraints and competing agendas, groups were limited to three, and this, unfortunately, prevented the participation of other regions, particularly EMTs and stakeholders coming from low- and middle-income countries; participants from these areas would have contributed enormously, particularly to the implementation and adaption of the framework, giving a wider perspective and securing a more inclusive exercise.

## 6. Conclusions

This study addresses a critical gap, contributes to defining surge capacity in the disaster medicine field, and advocates for the use of its components to strengthen health systems resilience, extendable to the configuration of deployable emergency medical teams.

This study introduces an innovative method by applying surge capacity components as pillars for structuring a framework for capacity-building efforts for disaster readiness and response, particularly in the context of the Emergency Medical Teams Initiative.

The framework presented in this study provides a unified approach to building response capacities, making it adaptable and replicable across a variety of contexts. Its systematic application can support standardization across countries and organizations, foster better outcomes during emergency responses, particularly in reducing morbidity and mortality, and enhance health workforce skills, leading to better management at the individual, organization, and community levels.

Finally, this study advocates for systematic and evidence-based improvements in the global disaster response capacity, emphasizing collaboration, standardization, and adaptability. This, in our opinion, will translate into the appropriate allocation of resources, improve disaster management and humanitarian interventions, and foster changes based on the context and the population in need. Since we are witnessing the challenges faced by health systems around the globe trying to cope with health emergencies, this framework is urgently needed.

## Figures and Tables

**Table 1:** Questions to focus group discussion participants.

1- What are your comments on the aim, objectives, and concepts shared in the preliminary capacity building framework?
2- What would successful implementation of the capacity building framework look like?
3- What are the roles of your organization in relation to the capacity-building pathways and how will you contribute to implement them?
4- Do you agree with the preliminary framework? And which recommendations would you provide to improve it?

**Table 2:** Key articles.

Manuscripts	Authors	Contributions
“Health Systems Surge Capacity”	K.Watson, J.W.Rudge and R. Coker. The Milbank Quarterly, Vol. 91, No. 1, 2013 (pp. 78–122) [16].	This paper is the first study to review in a comprehensive manner the emergence of the concept of surge capacity and its consolidation in the peer-reviewed and grey literature and assessed its extension at the policy level. Moreover, it is the first one to include in its discussion the concept of capacity building and presents the capacity building from Potter and Brough (2004) [23] as a subtype of surge capacity. Watson et al. proposed surge capacity as the portion of health system resources that can meet the surge needs from any kind of event. They observed the need for a conceptual and analytical framework to define the research for policy improvement and practice.
“Systematic Capacity Building: A Hierarchy of Needs”	Potter and Brough, 2004, [23]	The authors acknowledge the lack of a satisfactory definition of the term capacity building in the literature, even though it has been the objective of many development programs. They recognize as well that many undermine the term and confuse it with training. This paper provides an explanation of the term capacity building using a systems approach and identifies a pyramid of interconnected levels, forming the four-tier hierarchy of capacity building needs: (1) structure, systems, and roles; (2) staff and facilities; (3) skills; and (4) tools. The authors suggest that this systematic approach will improve the “diagnosis” of programs’ challenges and the design and monitoring of projects and will improve the appropriate use of resources.
“Health care facility and community strategies for patient care surge capacity”	Hick et al., 2004 [25].	This paper presents the areas in which expanded “surge” response might be required during a disaster: “public health surge capacity”, “healthcare facility-based surge capacity” and “community-based patient care”, whereby “surge capacity” is understood as the ability to manage a sudden and unexpected increase in the number of patients that would challenge the current capacity of the healthcare system. The authors suggest that the planning for the surge capacity should be modular, scalable, and flexible, and the strategies must be part of a comprehensive public health and emergency-management plan, allowing for the activation of multiple levels of capacities at the local/regional/national level.
“Understanding Surge Capacity: Essential Elements”	Barbisch and Koening, 2004. https://doi.org/10.1197/j.aem.2006.06.041 [26]	The authors acknowledge that there is not a definition of surge capacity and there is poor evidence-based planning for it. Moreover, they note that there are no validated measures to assess the effectiveness of surge-capacity interventions. They suggest four components for the surge capacity, which are known as the 4“S”: staff (trained personnel), stuff (comprehensive supplies and equipment), structure (facilities), and system (policies and procedures), noting that there should be linkages between these components to develop an optimal and sustainable surge capacity.
“Exploring the Concept of Surge Capacity”	Lavonne M. Adams. 2009 [24]	This paper agrees with previous literature on the lack of appropriate definition of surge capacity and it recognizes the imperative need to define the concept as it is essential for an effective disaster planning and preparedness particularly because it would facilitate its study and measurement. It observes that the term surge capacity is not static, but rather it has multiple components, and varies in different ways, depending on the demands of each component, and so does the organization’s surge capacity. The author proposed a definition for surge capacity as the “ability” to obtain adequate staff, supplies and equipment, structures, and systems to meet the needs following large-scale disaster.

**Table 3:** Similarities between surge capacity components and capacity building pyramid four-tier hierarchy.

	Capacity Building	Tools	Skills	Staff/Infrastructure	Systems and Roles
Surge Capacity					
Supplies		X			
Staff			X		
Structure				X	
Systems					X

**Table 4:** Concepts proposed to the three FGDs.

Capacity building	Any activity within the four S’s that is designed to improve or enhance the ability of one or more stakeholders from the EMT Network to absorb change effectively and sustain itself efficiently over time.
Surge capacity	Surge capacity refers to the ability of a healthcare system to alter its usual operations to accommodate increased patient volume due to an emergency or reduced healthcare capacity due to damage of existing facilities and/or a decrease in the available workforce.
EMT capability	The collective skills, expertise, and resources of an EMT organization to deliver on its deployment mandate according to the specified typology.
EMT coordination capacity	The mechanisms, processes, and skills necessary to activate the support of EMTs and seamlessly integrate their deployment within an affected healthcare system as temporary surge support.
Modalities	The actual activities by which stakeholders can feed into one or more capacity building pathways.

**Table 5:** Using the surge capacity components to build national and international teams’ capacities (framework).

4“S”	National EMTs	International Capacities	Transversal Activities
Staff	Training curriculum, taking into consideration the need for task shifting/repurpose/sharing. Team conformation based on context needs/healthcare services to be delivered/and availability of resources.	Conformation of teams based on international classification (EMT type I, II, III, and specialized care teams). Training curriculum (idem).	Recommended trainings: team members, trainer of trainings (ToT), Team leader, and ad hoc technical trainings. EMT’s training centers/hubs to foster knowledge sharing, strengthen communication channels, and bring a cohesive platform for skills sharing. Twinning project. On-the-job training.
Systems	Definition of standards based on context needs/configuration of national teams. Legislation: national clinical protocols. Ethical considerations. Adaptation of delivery of care to meet response needs. Governance.	SOPs as per Blue Book/Red Book. Clinical protocols of the country of deployment and in their absence to follow WHO recommendations. EMTCC/coordination mechanisms.	Coordination of national and/or international EMTs: EMTCC/EOC. Mobilization, deployment/receiving medical teams. Mentoring of teams, coaching and technical support. Adaptation of delivery of care to meet response needs. Twinning project.
Supplies and equipment	Development, purchase, and maintenance of medical and non-medical items, including equipment, consumables, and pharmaceuticals in line with country needs and policies. Reallocation of assets.	Development, purchase, and maintenance of medical and operational support items, including medical equipment, consumables, pharmaceuticals, and non-medical supplies in line with the EMT types and standards of care.	Pre-stored logistics and pharmaceuticals.
Structure/ Space	Repurposing/adaptation of existing facilities. Modular structures “ready to use” as a “stand-alone facility” or to “plug-in” to an existing one.	Configured structures according to EMT type.	Embed international staff into existing national structures.

## Data Availability

Data is contained within the article. Further inquiries can be directed to the corresponding authors.

## Abbreviations

ACLED	Armed conflict location and event data
ALNAP	Active learning network for accountability and performance in humanitarian action
CHS	Core humanitarian standard
CRIMEDIM	Center for research and training in disaster medicine, humanitarian aid and global health
COVID	Coronavirus infectious disease
EB	Executive board
EMT	Emergency medical teams
EMTCC	Emergency medical teams coordination cell
EOC	Emergency operations centre
FGD	Focus group discussion
IFRC	International Federation of the Red Cross
IHR	International Health Regulations
LMIC	Low–middle income countries
REGEDIM	Research Group on Emergency and Disaster Medicine
Swiss TPH	Swiss Tropical and Public Health Institute
ToT	Trainer of trainers
TOC	Theory of Change
UNFCCC	United Nations Framework for Climate Change
UNDRR	United Nations for Disaster Risk Reduction
WHO	World Health Organization

## Appendix A

**Table A1:** Focus group discussion participants.

Groups	Participants	Countries	Expertise
Group A	WHO EMT Regional Focal Points from PAHO/Regional Office at the America’s Regional Office EMRO Regional Office at the Easter Mediterranean Region EURO Regional Office SEARO Regional Office in Southeast Asia WPRO Regional Office in Western Pacific	United States Egypt Denmark India Philippines/Fiji	Health policy. Health emergencies, preparedness, and response.
Group B	WHO EMT Initiative Collaborating Centers: Karolinska University, CRIMEDIM-UPO, Sheba Medical Centre-Israel Centre for Disaster Medicine and Humanitarian Response, DG ECHO, HUG (Centre for Humanitarian Medicine and Disaster Management)	Sweden, Italy, Israel, Belgium, Switzerland	Research. Training-learning methodologies. Support to humanitarian operations. Data analysis
Group C	Representatives from Classified EMTs: UKMED, AUSMAT, UKEMT, Polish EMT, Italian (Regione Piemonte) EMT	United Kingdom, Australia, Poland, Italy	Health operations in a variety of contexts. Health strategy. Needs assessment, risk analysis, and program management.

**Table A2:** Examples of surge capacity definitions found in the literature.

Terms	Definitions	Reference/Authors
Surge capacity	“*Ability to manage a sudden, unexpected increase in patient volume that would otherwise severely challenge or exceed the current capacity of the healthcare system*”.	Hick et al., 2004 [25]
Surge capacity (proposed)	“*The ability to obtain adequate staff, supplies and equipment, structures, and systems to provide sufficient care to meet immediate needs of an influx of patients following a large-scale incident or disaster.* Acknowledging that there is the need to further refine it based on the type of surge-generating event, and stablishing areas of measurement of surge capacity”.	Lavonne M Adams, 2009 [24]
Surge capability	“*Ability of the healthcare system to manage patients who require specialized evaluation or interventions (e.g., contaminated, highly contagious, or burn patients)*”	Hick et al., 2004 [25]
Hospital surge capacity	“*Defined as the ability of a hospital to expand rapidly and augment services in response to one or multiple incidents, and to manage patients that require unusual or very specialized evaluation or intervention (e.g., contaminated or burn patients)*”	Djalali, Ingrassia, & Ragazzoni 2016. Ciottone’s Disaster Medicine Second Edition, 2016. [29]
Facility-based surge capacity	“*Actions taken at the healthcare facility level that augment services within the response structure of the healthcare facility; may include responses that are external to the actual structure of the facility but are proximate to it (e.g., medical care provided in tenting on the hospital grounds). These responses are under the control of the facility’s incident management system and primarily depend on the facility’s emergency operations plans*”.	Hick et al., 2004 [25]
Public health surge capacity	“*Ability of the public health system to increase capacity not only for patient care but also for epidemiologic investigation, risk communication, mass prophylaxis or vaccination, mass fatality management, mental health support, laboratory services, and other activities*”	Hick et al., 2004 [25]

**Table A3:** Definitions for capacity and capability building found in the literature.

Capacity building	“*The process of developing and strengthening the skills, instincts, abilities, processes, and resources that organizations and communities need to survive, adapt, and thrive in a fast-changing world*”.	United Nations [12]
	“*Capacity-building’ is a core concept of development policy. It is ‘planned development of (or increase in) knowledge, output rate, management, skills, and other capabilities of organization through acquisition, incentives, technology, and/or training*”.	European Parliament [13]
	“*In the context of transformation, capacity building refers to an individual or organization’s ability to absorb change effectively*”.	Caroline Kealy 2015 [30]
Capability building	“*Refers to the skills and knowledge required for a particular task. An organization may have the* capacity *to change but lack certain key capabilities*”.	Caroline Kealy 2015 [30]

**Table A4:** Theory of Change for the EMT Initiative capacity-building framework.

**Problem Statement** The increase in frequency, severity, and complexity of disasters and health-related emergencies combined with poor improvement in health and social indicators in low-and-middle-income countries (LMIC), and overall economic and financial imbalance across the world, are increasing health disparities and stretching the global humanitarian and health systems [31]. The confluence of these issues, the COVID pandemic, and the current and protracted conflicts have shown that the world is still ill-prepared to predict, prevent, respond to, and recover from multi-country outbreaks and other disasters. **Hypothesis** Enhancing surge capacity supports readiness and rapid deployment of EMTs (emergency medical teams), enabling Member States and organizations to mitigate the impact of health emergencies.			
**Inputs**	**Activities**	**Outputs**	**Outcomes**
(1) EMT Secretariat and regional offices staff (2) Emergency medical teams (3) Donors (4) Member States (5) WHO collaborating centers (6) Training hubs (7) Humanitarian organizations and other stakeholders	(1) Contextualization of the training pathway. (2) Revision of the operational learning and research framework. (3) EMTs Training Hubs (4) Training of EMTs Coordination Cell (EMTCC) personnel. (5) Develop standards and guidelines for EMTs based on risk analysis and contexts needs. (6) Foster collaboration with academic institutions and worldwide medical associations. (7) Continued classification of emergency medical teams. (8) Multistakeholder engagement and partnerships. (9) Information sharing and diplomatic engagement with donors. (10) Improve program performance, monitoring, and evaluation.	(1) Training pathway is developed and strengthen to ensure EMTs capabilities readiness. (2) Coordination mechanism (EMTCC) is timely and efficiently activated to support Member States and EMTs responses. (3) Clinical, technical, and operational standards are developed to guarantee best practices and the delivery of high-quality care. (4) National and International EMTs classification is establish through a qualitative process to secure a multidisciplinary global emergency health workforce. (5) Regional and global partnerships are expanded fostering participation of Member States and reinforcing wider cooperation. (6) Community of practice and knowledge sharing platforms are strengthened to develop evidence-based strategies through research and deep contexts analysis.	(1) Emergency preparedness, readiness and response at local, national, and international level is strengthen and improved. (2) Global capacity and capability are empowered through the adoption and implementation of emergency medical teams standards of care. (3) Rapid mobilization of medical teams and efficient coordination are reinforced. (4) Health workforce skills and knowledge are enhanced. (5) EMTs infrastructures are flexible, modular, and rapidly positioned where they are needed.
**Impact** Enhance surge capacity of Member States through rapid mobilization and coordination of national and international emergency medical teams (EMTs); therefore, loss of life and long-term disability caused by disasters, outbreaks, and other complex emergencies will be prevented and mitigated.			
